# Trade-offs between short- and longer-term resilience to warming within and between subtidal marine assemblages

**DOI:** 10.1038/s41598-025-07457-w

**Published:** 2025-07-02

**Authors:** Jesse M. A. van der Grient, Gareth Waters-Price, Barend Stander, Paul Brickle, Simon A. Morley

**Affiliations:** 1 South Atlantic Environmental Research Institute, Stanley, Falkland Islands; 2 School of Biological Sciences, University of Portsmouth, Portsmouth, UK; 3 Falklands Fish Farming (Ltd), Stanley, Falkland Islands; 4 School of Biological Sciences, University of Aberdeen, Aberdeen, UK; 5 British Antarctic Survey, National Environment Research Council, Cambridge, UK

**Keywords:** Ecological traits, Environmental predictability, Falkland Islands, Latitude, Marine ectotherms, Coastal assemblages, Climate-change ecology, Community ecology, Ecophysiology, Animal physiology

## Abstract

Subtidal marine ectotherm physiological responses vary with ocean warming. Predicting these responses is important for ecosystem assessments to inform management and conservation strategies. Falkland Islands coastal species representing different mobility, feeding guilds, and habitats were tested, through laboratory incubation experiments, to estimate their short- (acute - seconds to hours) and longer-term (acclimation - weeks to months) resilience to ocean warming, to understand if ecological traits affect temporal trade-offs in responses, and contrasted with other marine assemblages. We found trait-specific, and species-specific, trade-offs in resilience to short-term and longer-term warming. Filter feeders and predators had higher acute tolerance than detritivores and herbivores. Lower acclimation capacity was found in molluscs, sessile species, filter feeders and kelp associated species. Benthic species had amongst the highest acclimation capacity. When compared to analogous experiments conducted with the same methodology at 10 different locations, across latitudes, we found a consistent relationship between short- and long- term resilience across marine assemblages, but with notable exceptions from unpredictable environments with episodic warming events; the Peruvian upwelling and Falklands fauna had a lower short-term resilience, relative to their longer-term resilience, than the other assemblages. When predicted rates of ocean warming under a high anthropogenic carbon emission scenario and anticipated increases in marine heat waves were taken into account, low latitude assemblages showed greater vulnerability in terms of years until acute thermal safety margins are breached (less than 500 years) than higher latitude assemblages (up to 4000 years), which is largely driven by projected rates of ocean warming. Understanding this variation, and the relationship to predictability, in coastal communities will be informative for predicting ecosystem responses and informing management and conservation strategies.

**Supplementary Information:**

The online version contains supplementary material available at 10.1038/s41598-025-07457-w.

## Introduction

Subtidal marine biological communities across the world are changing in response to climate change, with implications for ecosystem functioning and services. While multiple environmental factors, varying over a range of scales, affect animal physiology, temperature is one of the most significant as it affects the rates of all ectotherm physiological processes^1,2^. The effects of temperature on biological rates are species specific and thermal performance responses will, therefore, affect the physiological resilience of species to ocean warming. Thermal stability is one of the key environmental factors that is correlated with ectotherm resilience to warming, as evidenced through latitudinal comparisons^3–7^. Understanding the effects of different dimensions of ocean warming (e.g., mean temperature rise, extreme events, seasonality) on biological rates, and the implications for the functioning of animal assemblages is vital as we aim to understand wider ecological responses to climate change. This understanding is crucial to improve ecosystem models that provide the basis for ocean management.

Testing animal resilience to ocean warming—the maximum temperature that can be tolerated before loss of equilibrium (CT_max_) and potential mortality—requires experimental assays at ecologically relevant scales^8^. Experiments utilising rapid rates of warming (short term - seconds to hours), which can predict response to marine heat waves (MHW), are not necessarily good predictors of likely response to climate change (longer term—weeks to months). Heating assays that test species at different rates of warming are one technique that allows extrapolations from laboratory experiments to ecologically relevant rates of warming^6^. Dynamic assays, where animals are heated at constant rates until collapse, demonstrate that under simulated MHW, many animal taxa have higher whole-animal thermal limits (CT_max_; critical thermal maxima) than during slower rates of warming^6,9–12^. Further, assemblages with higher CT_max_ tend to have shallower slopes, indicating they have a greater capacity for tolerating change^11^. These estimates are influenced by various factors, such as acclimation capacity, speed of acclimation^13^, species mobility^14,15^, experimental protocols^16–18^, and evolutionary history, including in response to environmental predictability^15,17,19^. Estimating these relationships allows predictions of animal resilience to climate warming scenarios, which cannot be replicated under laboratory conditions^6,11^.

The Falkland Islands are a cold temperate island archipelago in the southwest Atlantic Ocean, with a high diversity within the nearshore kelp forest ecosystem. The offshore waters surrounding the Falklands are typical of high latitude regions that border the Southern Ocean, with low population densities, low levels of anthropogenic pollution, but a long history of ecosystem exploitation. Many species that live offshore as adults, including commercially exploited species, use the kelp forest as a nursery habitat, making this an important habitat for the diversity and economy of the Islands^20^. The wider southwest Atlantic Ocean has seen increases in sea surface temperature (SST), and MHW frequency and duration over the last four decades^21–23^. Most of this regional warming has occurred to the north of the Falkland Islands, where the Falkland and Brazilian Currents flow eastwards between the Sub-Antarctic and Sub-tropical fronts^24^. The Falklands offshore areas largely remain bathed in the cold northward flowing Falkland Current, whose SST has either not changed or reduced slightly in temperature depending on the models used, thus keeping the Falklands offshore thermal environment relatively stable, with one of the lower rates of global warming^21,22,25–27^. However, air temperatures have increased over the Falkland Islands, and nearshore kelp forest areas could be warming. Consequently, there is a need to understand how soon current rates of warming are likely to affect animal communities living in or around the Falklands kelp forests to inform the potential consequences for the wider food web because of the ontogenetic and trophic linkages to offshore areas^28^. The understanding gained about the resilience of the Falklands ecosystem to warming, is likely to provide insights for other similar high latitude regions.

Temperature will not increase in isolation, as changes in ocean currents will likely also result in changes in nutrients, oxygen, salinity and pH^29^. Air temperature is also just one of the factors that is changing with climate, for example patterns of precipitation are also changing^30 ^which will alter surface salinity and the amount of incident light. It is also not just mean climate that is changing but also extreme events are becoming more frequent and more intense^31^. However, temperature affects all the biochemical reactions of ectotherms making it a key environmental factor determining ectotherm physiology and therefore species resilience^32^.

As of yet, no physiological experiments have been conducted in the Falkland Islands and so there is no understanding of the vulnerability of kelp-associated and non-kelp-associated animals to coastal warming. Here, we investigate the response to different rates of warming of selected animal species, representing key groups within the nearshore marine assemblage (Mollusca, Arthropoda, Echinodermata). We hypothesize that differences in tolerances are species specific, and that species traits, such as mobility, habitat, feeding guild, and association with kelp forests, will influence the level of temperature variability animals naturally experience, and therefore their evolved physiological resilience. The presence of kelp reduces the mixing of water, thereby potentially leading to more locally stable temperatures within, compared to outside, the kelp forests^33^. The natural environmental variability experienced by species will also depend on their habitat depth, with near surface pelagic species possibly experiencing different temperatures than subtidal benthic species. Mobile species also have the option to move away from damaging temperatures, unlike sessile species who are fixed to the substratum^34^. Pelagic species have greater opportunities to move than benthic species and this could mean that they are able to redistribute to avoid heat extremes, theoretically leading to them evolving a reduced tolerance compared to species that are constrained to a particular environment and therefore have to tolerate their experienced extremes, particularly in the intertidal^35^. Alternately, active species typically have a higher aerobic scope and can therefore tolerate higher temperatures than less active species, as per the expectation of the hypothesis of oxygen and capacity limited thermal tolerance^2,36–38^. The aim was to provide an indication of the influence of habitat and functional metrics on warming responses, whether there are trade-offs in short- and longer-term responses and how the resilience of Falklands species compares to other marine assemblages. We further hypothesize that the community response of the Falkland Islands will be in line with responses across latitudes^11^.

## Materials and methods

### Collection

**Fig. 1:**
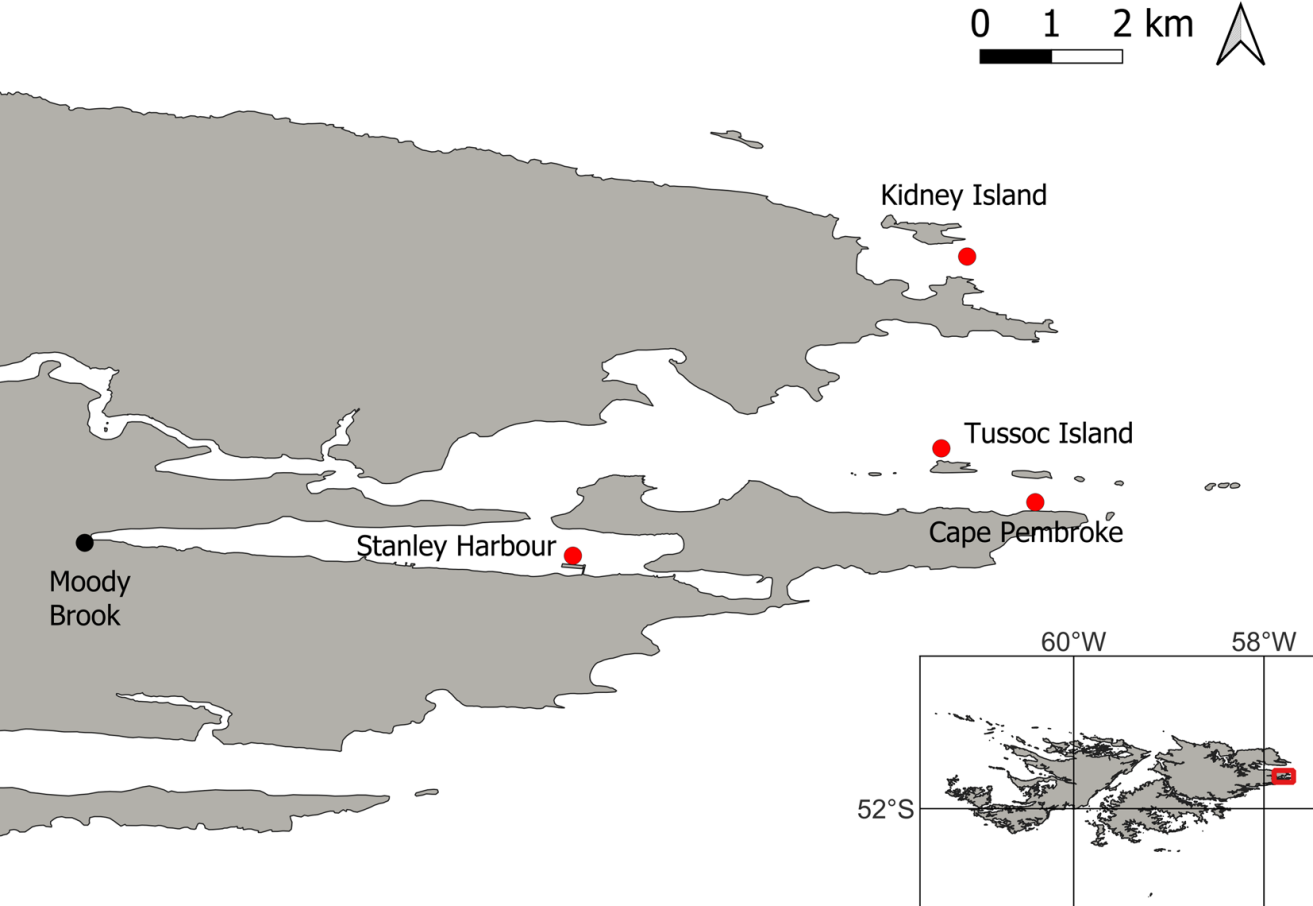
Sample (red circles) and experimental facility (black circle) locations. Inset shows the Falkland Islands with the red rectangle indicating the sample area on the East Falkland coast.

Invertebrates living on or near kelp forests in the Falkland Islands were collected. Species collected by SCUBA divers (10–20 m depth) and by snorkellers (< 2 m depth), when sea-surface temperature generally vary between 9.5 and 10.5 °C, but can be as low as 7 °C (October (spring) in 2022; off Kidney Island (Fig. 1)) or as high as 11.6 °C (February (summer) in 2023; off Kidney and Tussock Islands (Fig. 1)). Species collected included the leaden whelk (*Pareuthria plumbea*), pink pencil urchin (*Austrocidaris canaliculata*), scythe-edged serolis (*Acanthoserolis schythei*), kelp limpet (*Nacella mytilina*), kelp isopod (*Cassidinopsis emarginata*) and kelp bivalve (*Gaimardia trapesina*). Two size classes of lobster krill (*Grimothea gregaria*) were included: small *G. gregaria* were caught using scoop nets as they were swarming on the surface in one of the local harbours, and large *G. gregaria* were caught using a small dredge. Small *G. gregaria* are predominately pelagic in the wild, although in the tanks they settled on the bottom, while the large *G. gregaria* live a benthic lifestyle. Species were collected during Spring and Summer in 2022 around East Falkland Island, from Kidney Island, Tussock Island, Cape Pembroke, and east Stanley Harbour (Fig. 1). Species were transported to the Falklands Fish Farming Ltd aquacultural facility located in Moody Brook, which was the only flow-through seawater facility in the Falklands.

### Set up

Seawater for the facility was pumped from the west of Stanley Harbour after which it was treated with UV light, ozone, and large particles removed via a sand stiller and a protein skimmer before entering the header tank. This water was re-circulated within the experimental system from the header tank with roughly 10% of the water in the system being replaced each day. The experimental tanks were connected to a drain to ensure warmed water did not re-enter the re-circulating system. During trials, animals were fed with kelp, pieces of fish or squid to satiation. *G. trapesina*, *C. emarginata*, and small *G. gregaria* were kept in small containers within a larger tank to ensure they could be tracked and to prevent predatory interactions (*P. plumbea*, for example, predate on *N. mytilina*). Others, such as the large *G. gregaria*, *A. schythei*, *P. plumbea* and *A. canaliculata* could be kept together without risk of negative interactions. Day-night cycles were created by manually switching lights on at 8 am, and off at 5 pm. Natural light could still enter the building before or after these times, but light levels were very dim, generating a natural photoperiod.

There is no temperature-controlled room available in the Falkland Islands, and daily temperature checks showed that the ambient temperature in the control tanks could vary several degrees, especially during the long-term experiments. Control tanks were monitored to check for any adverse effects of culture conditions (Table S1). Animals were introduced into tanks and allowed a minimum of 14 days to adjust to control conditions and to monitor for any mortality. Small *G. gregaria* initially suffered several deaths in the control tank during this adjustment period, prior to the commencement of the experiment because of conspecific attacks which occurred during or after moulting, even when food was present. This was addressed by spreading the *G. gregaria* over more small containers floating in larger tanks to reduce densities in containers.

Organisms were kept in tanks of 900 L, and experimental tanks were heated by 2KW PRO-Line titanium heaters controlled by a 0.1 °C precision digital thermostat. Temperatures in experimental tanks were raised at three different rates: 0.04 d °C^− 1^ (1 °C hour^− 1^), 1 d °C^− 1^ (1 °C day^− 1^), 7 d °C^− 1^ (1 °C week^− 1^). Experiments took a maximum of 20 h (0.04 d °C^− 1^), 20 days (1 d °C^− 1^), and 19 weeks (7 d °C^− 1^) to run, whereby starting water temperatures ranged between 9.5 and 11.5 °C, and were limited to a maximum of 30 °C. Space and the availability of heaters and temperature probes put a limit on the number of experimental tanks we could run at the same time, which was two, with one control tank. Because of logistical challenges, we ran two different experiments at the same time (0.04 d °C^− 1^, 1 d °C^− 1^), with the control tank running continuously while any experiment lasted. The 0.04 d °C^− 1^ and 1 d °C^− 1^ experiments ran between 30 December 2022 and 17 January 2023, while the 7 d °C^− 1^ experiment ran from 10 March 2023 to 9 June 2023.

Fitting a regression relationship to CT_max_ measured at different rates of warming allows short-term (hours to days) and longer-term (weeks to months) thermal tolerance to be calculated and long-term resilience to be extrapolated. The upper temperature limits of animals (CT_max_) were determined by tactile stimuli (*P. plumbea*, *A. schythei*, and *G. gregaria* were stimulated with a blunt seeker), or visual observation (movement for *A. canaliculata*, *P. plumbea*, *G. gregaria*, and *A. schythei*). Animals that failed to stay attached to the sides of the tanks (*N. mytilina*, *G. trapesina*) were stimulated with a blunt seeker to illicit a response. The lack of response was recorded as CT_max_. Mortality was assessed every hour in the 0.04 d °C^− 1^ treatment, twice a day in the 1 d °C^− 1^ treatment and every day in 7 d °C^− 1^ treatment. Temperatures were not raised above 30 °C as per heater manufacturer recommendation. Ethical approval for collecting and using animals in the experiments was obtained under research license number R08/2022 from the Falklands Islands Government review board, and experiments were performed in accordance with these guidelines.

### Statistical analyses

Relationships between rate of warming and CT_max_ were investigated using linear models. CT_max_ was natural log transformed to meet model assumptions of homogeneity of variance and normality of residuals. Several linear models were constructed to investigate the categorical factors affecting CT_max_: (M1) the interactive effect of species with rate of warming; (M2) interactive effect of taxon (Crustacean, Mollusc, or Echinoderm) with rate of warming; (M3) interactive effect of mobility (sessile, low mobility, high mobility) with rate of warming; (M4) interactive effect of feeding guild (filter feeders, detritivores, herbivores, predators) with rate of warming; (M5) interactive effect of habitat (benthic, pelagic, on the kelp-associated) with rate of warming; and (M6) interactive effect of kelp forest (inside or outside) with rate of warming. Model diagnostic plots were inspected to determine whether model assumptions (residuals are normally distributed, equal variance in residuals) had been met. The overall significance of the interaction between the grouping variable and rate of warming was tested using a Chi^2^ test (with Deviance (D) as test statistic). See Table 1 for the categorisation of each species. Results were considered statistically significant when p-values were lower than the threshold $\:\alpha\:=0.05$. Given that a number of models are tested, the p-values were adjusted using the corrections by Benjamini & Hochberg^39 ^also known as BH or fdr correction. This method reduces type I error and minimizes type II errors.

A linear mixed effect model was constructed to investigate the relationship between rate of warming and CT_max_ in different assemblages. Data were taken from previous studies using the same methodologies, where different rates of warming were used (summarised in Morley et al., 2014). The assemblages compared were from the Antarctic Peninsula, McMurdo Sound Antarctica, Scotland, New Zealand, shallow (< 2 m) warm temperate France and the US, warm temperate France and the US, warm temperate Peru, St Helena, Ascension Island and Singapore. Both CT_max_ and rate of warming were natural log transformed, following Richard et al. (2012) and Morley et al. (2014), in order to extract and compare the intercept and slope values of different assemblages. Species was included as a random variable in the model to construct a random-slope, random-intercept model, accounting for the non-independence of taxa.

To understand the warming tolerance between the acute CT_max_ and currently experienced temperature of each assemblage we calculated the difference between CT_max_ and the maximum monthly sea surface temperature at each location (http://www.seatemperature.info). We then calculated the predicted annual rate of warming of that same location (calculated using SSP5-8.5 experiments, the average of all models, ensemble spread of future change between the periods 1955–1984 and 2020–2049; http://www.psl.noaa.gov/ipcc/cmip6). By dividing the average warming tolerance by the projected warming, we were able to estimate the number of years until CT_max_ would be breached. To understand how MHW would change this estimate, we reran this analysis and included the estimated elevation of MHW for each specific location, taken from the marine heatwave forecast monthly report (https://psl.noaa.gov/marine-heatwaves/#report). These analyses were performed for a subset of the locations included in the linear mixed effect model for which data on individual species were available. A second-order polynomial model was fitted to the data to determine whether there is a relationship between number of years until CT_max_ is breached and latitude. The model was fitted with an interaction between latitude and the presence or absence of marine heatwaves to understand if this affects the relationship. Using the high emissions SSP5 – RCP 8.5 models give a “worst-case” scenario, in terms of years of resilience, but the relative predictions will remain the same even if lower emission scenarios are used.

Data analyses and visualisation were analysed using R version 4.1.2^40^. The package nlme^41^ was used to construct the mixed effects model. The package ggplot2^42^ was used to visualise the results.

## Results

### Falkland Islands assemblage CT_max_

CT_max_ differed between species and with the rate of warming (Table 1). CT_max_ ranged from 30 °C (*P. plumbea* at 0.04 d °C^− 1^) to 13 °C (*C. emarginata* at 7 d °C^− 1^). *N. mytilina* were the least temperature tolerant (18 °C) at 1 d °C^− 1^. 2 out of 7 *A. schythei* and 3 out of 7 *P. plumbea* reached CT_max_ in the 0.04 d °C^− 1^ treatment before 30 °C was reached. There was an opportunity to rerun the *P. plumbea* experiment a month later, when all individuals reached CT_max_, but this opportunity was not possible for the *A. schythei*. It was therefore not possible to determine the CT_max_ for *A. schythei* in the 0.04 d °C^− 1^ treatment but the data for the second trial of *P. plumbea* were used in further analyses. For all other species, 100% of individuals reached CT_max_ before 30 °C water temperature was reached. *A. schythei* were the most temperature tolerant in the 0.04 d °C^− 1^ and 1 d °C^− 1^ treatments, while the large *G. gregaria* were the most temperature tolerant in the 7 d °C^− 1^ treatment (Table 1). Small *G. gregaria* were the least temperature tolerant in the 0.04 d °C^− 1^ treatment, while showing high tolerance in the other two warming treatments.

**Table 1:** Rate of warming (Rate), number of individuals (N) used in each treatment, median CT_max_ (and interquartile range (IQR)) for each species in treatment, and starting temperature of the water (T_start_).

Species	Scientific name	Rate	*N*	CT_max_ (IQR)	T_start_	Taxon	Mobility	Guild	Habitat	Forest
Small lobster Krill	*Grimothea gregaria*	°/hour	15	26 (0.75)	10.5	C	H	D	P	O
		°/day	12	21 (1.75)	10					
		°/week	9	16 (1.0)	11.5					
Large lobster Krill	*Grimothea gregaria*	°/hour	8	23 (0.25)	10	C	H	D	B	O
		°/day	8	23 (0)	9.5					
		°/week	7	24 (0)	11.5					
Scythe- edged serolis	*Acanthoserolis schythei*	°/hour	7	na	10.5	C	H	D	B	O
		°/day	7	28.5 (1)	10					
		°/week	7	19 (4)	11.5					
Pink pencil urchin	*Austrocidaris canaliculate*	°/hour	10	26 (1)	10.5	E	L	H	B	I
		°/day	10	19 (3.63)	10					
		°/week	7	20 (1)	11.5					
Kelp limpet	*Nacella mytilina*	°/hour	8	29 (2)	10	M	L	H	K	I
		°/day	8	18 (2)	10					
		°/week	8	15.5 (5.25)	11.5					
Leaden whelk	*Pareuthria plumbea*	°/hour	9	30 (1)	10, 10.5	M	L	P	K	I
		°/day	8	23 (1)	10					
		°/week	7	20 (0.5)	11.5					
Kelp bivalve	*Gaimardia trapesina*	°/hour	13	28.5 (1)	10.5	M	S	F	K	I
		°/day	9	19.5 (1)	9.5					
		°/week	7	14 (1)	11.5					
Kelp isopod	*Cassidinopsis* *emarginata*	°/hour	7	24 (1.5)	10	C	H	H	K	I
		°/day	5	19.5 (0)	9.5					
		°/week	11	13 (5.5)	11.5					

Note that *P. plumbea* has two starting temperatures, reflecting two trials. Classifications are as follows: taxon: C = Crustacean, E = Echinoderm, M = Mollusc; mobility: S = Sessile, L = low mobility, H = high mobility; Feeding guild: D = Detrivore, H = Herbivore, F = Filter feeder, P = Predator; Habitat: P = Pelagic, B = Benthic, K = Kelp-associated; and Forest: O = outside, I = inside. na = not available.

### Relationships between functional groups

Rate of warming had a consistent significant effect on CT_max_ (D = 4.50, *P* < 0.001) and significant interactions with factors, species phyla, mobility, feeding guilds, habitat, and whether species live inside or outside kelp forests, in each model.

**Fig. 2:**
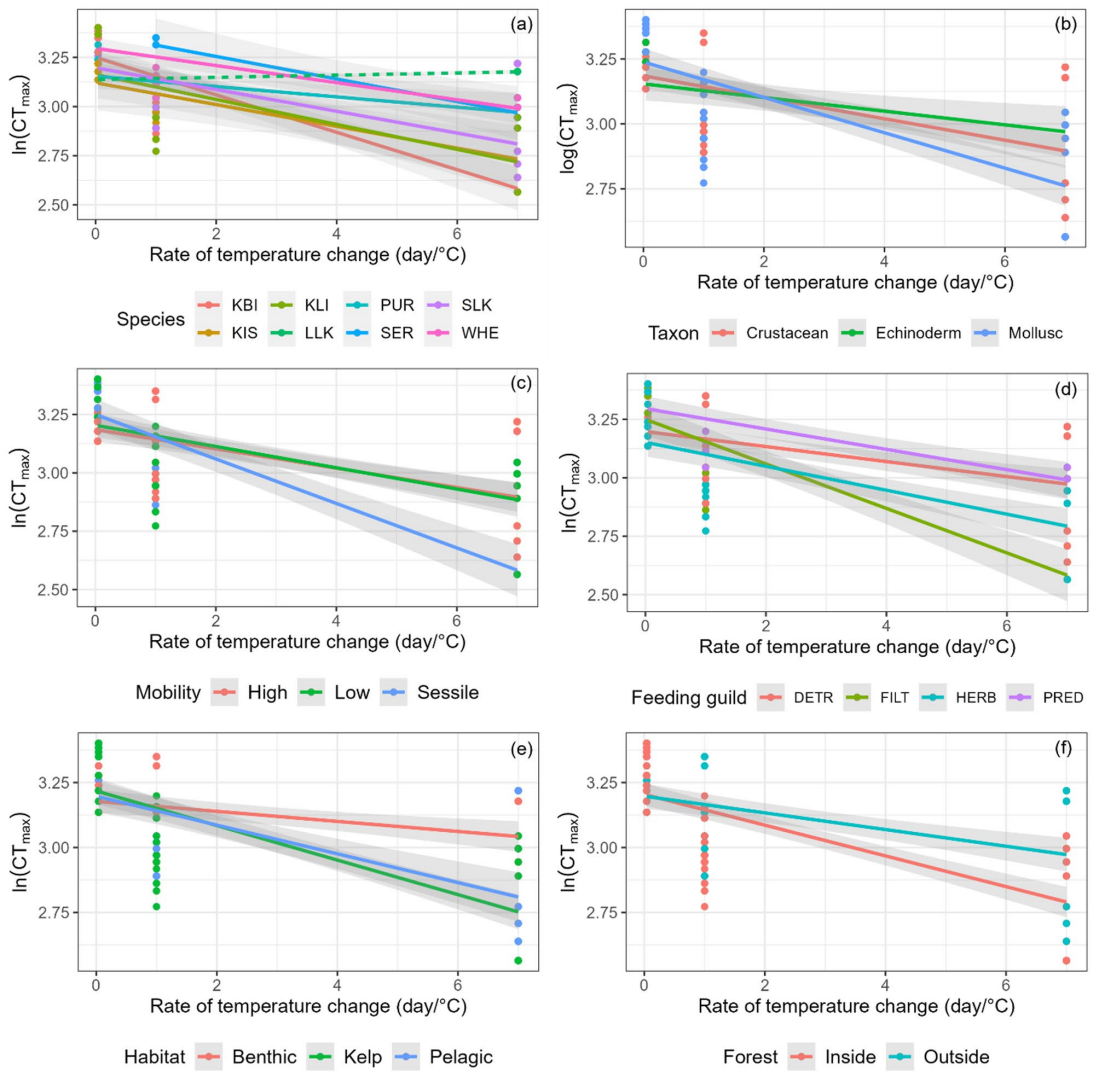
Relationship between rates of warming and CT_max_ for the Falklands faunal assemblage (**a**) by species (KBI = *G. trapesina*, KLI = *N. mytilina*, PUR = *A. canaliculata*, SLK = small *G. gregaria*, KIS = *C. emarginata*, LLK = large *G. gregaria*, SER = *A. schythei*, and WHE = *P. plumbea*), (**b**) by phyla, (**c**) by mobility, (**d**) by feeding guild (DETR = detritivore, FILT = filter feeder, HERB = herbivore, PRED = predator), (**e**) by habitat, and (**f**) by association with the kelp forest. Solid lines indicate significant relationships, while dotted lines indicate non-significant relationships.

Species (M1): CT_max_ was significantly different between species (D = 1.11, *P* < 0.001), and there was a significant interaction, with species specific responses to the rate of warming (D = 1.32, *P* < 0.001; Fig. 2a; Table 2). *G. trapesina* had the greatest drop in CT_max_ (steepest slope) with the rate of warming (reduced longer-term resilience), followed by a group of species with roughly similar rates of decline (*N. mytilina*,* A. schythei*,* C. emarginata*, and small *G. gregaria;* Fig. 2a; Table 2). *A. canaliculata* had the smallest difference in CT_max_ between different rates of warming (shallowest slope), while large *G. gregaria* had the same CT_max_ irrespective of the warming rate, so no difference between short-term and longer-term tolerance (no significant slope). Tolerance to acute warming (intercept) differed between species, and *A. schythei* and *P. plumbea* were more tolerant of MHW, while *C. emarginata* and large *G. gregaria* were less tolerant.

Phyla (M2): CT_max_ was not significantly different between phyla (D = 0.01, *P* > 0.05), although taxon and rate of warming had a significant interaction (D = 0.42, *P* < 0.01; Fig. 2b; Table 2). The mollusc group had a steeper slope, implying a lower longer-term capacity compared to the crustacean and echinoderm groups which did not differ from each other.

Mobility (M3): CT_max_ was not significantly different due to mobility alone (D = 0.07, *P* > 0.05), but mobility had a significant interaction with rates of warming (D = 0.56, *P* < 0.001; Fig. 2c; Table 2). Both the low and high mobility groups showed a similar response to rate of warming, while the sessile group showed a reduced capacity to cope with slower rates of warming (acclimation). Their responses to acute warming did not differ, however, as indicated by the similar intercept values.

Feeding Guilds (M4): CT_max_ was significantly different between feeding guilds (D = 0.68, *P* < 0.001) and there was a significant interaction between feeding guild and rate of warming (D = 0.70; *P* < 0.001; Fig. 2d; Table 2). Filter feeders had a significantly lower longer-term tolerance than the other three feeding guilds. Tolerance to acute warming was higher in filter feeders and predators than herbivores.

Habitat (M5): CT_max_ was significantly different between habitats (D = 0.30, *P* < 0.01) and there was a significant interaction between habitat and the rate of warming (D = 0.82, *P* < 0.001; Fig. 2e; Table 2). Species from benthic habitats had a shallower slope with rate of warming compared to pelagic or kelp-associated species, which had similar capacities. The tolerance of acute warming was similar between the three groups.

Kelp forest association (M6): CT_max_ was significantly different between kelp forest and non-kelp forest species (D = 0.18, *P* < 0.05) and there was a significant interaction between association with kelp forest and the rate of warming (D = 0.31, *P* < 0.01; Fig. 2f; Table 2). Species found inside the kelp forest had reduced longer-term tolerances, although the intercept between the two groups did not differ.

**Table 2:** Estimates for each tested variable in their respective linear models.

Model: variable	Categorical variable	Intercept (SE)	Slope (SE)
M1: species	*G. trapesina*	3.250 (0.034)^a^	− 0.195 (0.010)^a^
	*N. mytilina*	3.163 (0.041)^ab^	− 0.064 (0.010)^b^
	*A. schythei*	3.369 (0.067)^a^	− 0.057 (0.013)^bc^
	*C. emarginata*	3.121 (0.046)^b^	− 0.055 (0.010)^bc^
	Small *G. gregaria*	3.197 (0.031)^ab^	− 0.055 (0.009)^bc^
	*P. plumbea*	3.296 (0.039)^a^	− 0.044 (0.010)^bcd^
	*A. canaliculata*	3.154 (0.037)^ab^	− 0.026 (0.010)^cd^
	Large *G. gregaria*	3.137 (0.041)^b^	0.006 (0.010)^d^
M2: taxon	Mollusc	3.239 (0.026)^a^	− 0.068 (0.007)^a^
	Crustacean	3.185 (0.025)^a^	− 0.041 (0.006)^b^
	Echinoderm	3.154 (0.043)^a^	− 0.026 (0.012)^b^
M3: mobility	Sessile	3.250 (0.040)^a^	− 0.095 (0.011)^a^
	Low	3.204 (0.026)^a^	− 0.046 (0.007)^b^
	High	3.185 (0.024)^a^	− 0.041 (0.006)^b^
M4: feeding guild	Filter feeder	3.250 (0.038)^a^	− 0.095 (0.011)^a^
	Herbivore	3.151 (0.026)^b^	− 0.051 (0.006)^b^
	Predator	3.296 (0.043)^a^	− 0.044 (0.011)^b^
	Detrivore	3.197 (0.025)^ab^	− 0.032 (0.006)^b^
m5: habitat	Kelp	3.218 (0.022)^a^	− 0.066 (0.005)^b^
	Pelagic	3.197 (0.035)^a^	− 0.055 (0.010)^b^
	Benthic	3.178 (0.028)^a^	− 0.019 (0.007)^a^
M6: Kelp forest	Inside	3.204 (0.020)^a^	− 0.059 (0.005)^a^
	Outside	3.197 (0.027)^a^	− 0.032 (0.007)^b^

SE = standard error. All slope values, except for large *G. gregaria* were significantly different from 0. Subscript letters indicate similarity between intercepts (acute tolerance) or slopes (relative longer-term tolerance) within each variable.

### Global comparison—trade-offs between short- and long-term resilience

**Fig. 3:**
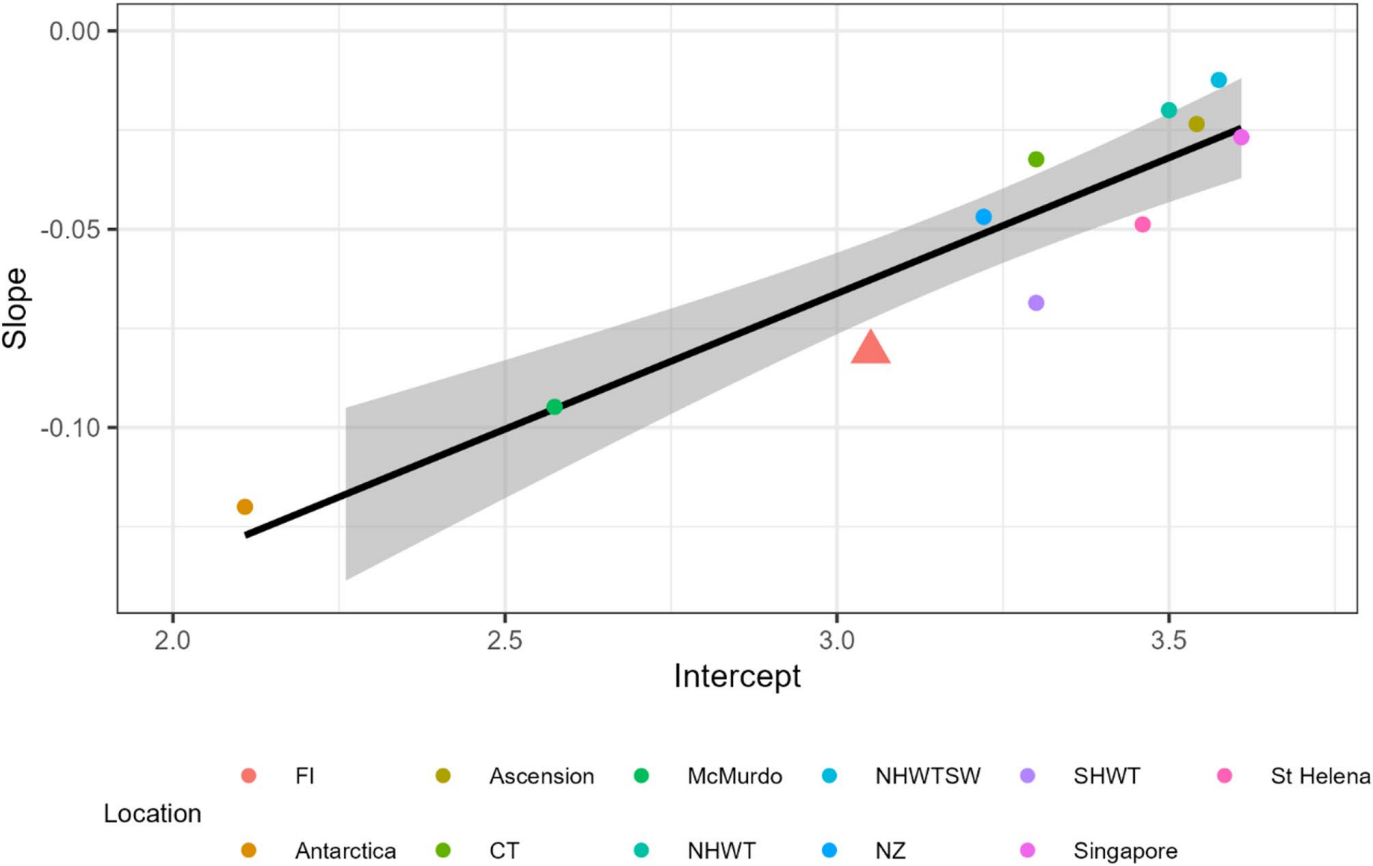
Slope and intercept values representing relationship between rates of warming and CT_max_ based on community assemblages from different locations, with 95% confidence intervals (grey shading). Data from locations other than the Falkland Islands are extracted from Morley et al. (2014) and Richard et al. (2012). FI = Falkland Islands; CT = cold temperate (Scotland); NHWTSW = < 2 m depth warm temperate (France, US); SHWT = warm temperate (Peru); NHWT = warm temperate (France, US); and NZ = New Zealand. The Falkland Islands is highlighted by the triangle shape and larger size.

Overall, there is a significant (F = 55.1, *P* < 0.0001) positive relationship between intercept and slope values, where assemblages that have higher intercept values also have shallower slope values (intercept: -0.271 ± 0.030 standard error (SE); slope: 0.068 ± 0.009 SE). The results from the mixed effect model demonstrated that the Falklands faunal assemblage fell below the predicted line for the assemblages, indicating a more negative slope than expected for the intercept value (Fig. 3). The intercept for the Falklands assemblage was most similar to the New Zealand assemblage, another cold temperate environment, and the Peruvian upwelling assemblage (SHWT in Fig. 3), a warm temperate environment.

### Projected resilience to warming and heatwaves

**Fig. 4:**
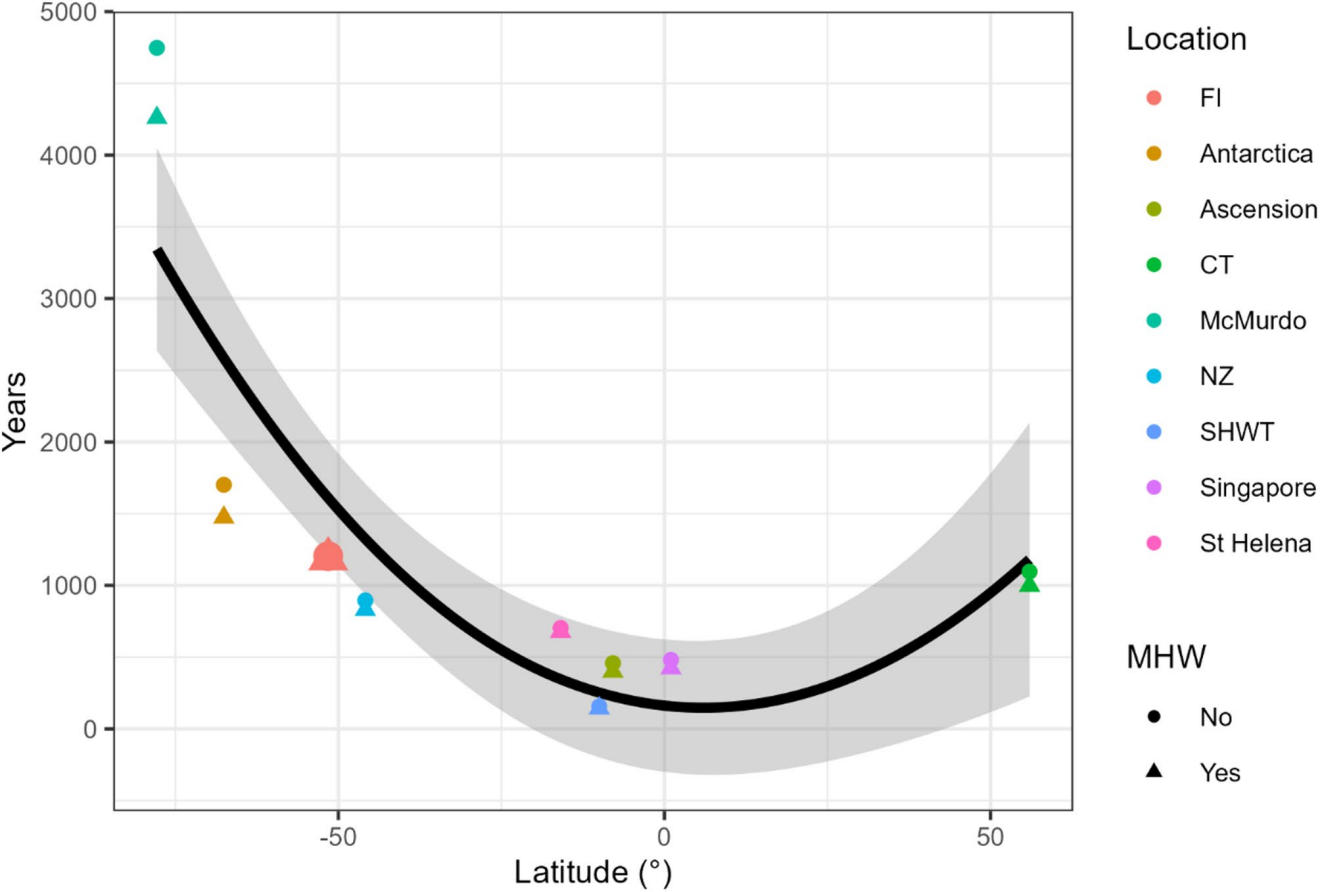
The number of years it will take before the CT_max_ of an assemblage is reached against their latitude. The different colours represent different locations. FI = Falkland Islands; CT = cold temperate (Scotland); SHWT = warm temperate (Peru); and NZ = New Zealand. The different shapes refer to whether the MHW elevation was applied in the estimate of the number of years until CT_max_ would be reached or not. The Falkland Islands is highlighted by the triangle shape and larger size.

The number of years until acute CT_max_ is breached differed between locations, with the McMurdo assemblage having the longest temporal buffer before temperatures that match CT_max_ are reached (> 4000 years). The Peruvian (SHWT) assemblage had the shortest temporal buffer (< 160 years; Fig. 4). The inclusion or exclusion of MHW temperature elevation affected the assemblages differently, with heat waves causing the greatest reduction in temporal buffer (487 years) for the McMurdo assemblage, while Peru was least affected by the inclusion of heat waves (14 years). Note that the CT_max_ for several species from the Peruvian assemblage was lower than the maximum monthly temperature, and this number was increased when the MHW elevation was included. The Falkland Islands assemblage would take between 1185 years (with MHW elevation included) to 1204 years (without MHW elevation included) for temperatures matching CT_max_ to be reached, and like Peru, the difference between the years with MHW elevation and without was low (19 years).

The highest latitudes generally had a higher estimate for the temporal buffer before CT_max_ was reached compared to lower latitudes. Both the interaction between latitude and the presence or absence of marine heatwaves and the additive effect of the presence of marine heatwaves was not significant (*P* > 0.05) and these were sequentially removed from the model. Latitude was the only significant factor that was included in the final second-order polynomial describing the years until CT_max_ was reached. Both the intercept (764.8 years ± 298.8 (standard error)) and the relationship (− 18.4° ± 6.5) were significant (*P* < 0.05).

## Discussion

### Falkland Islands assemblage

In this study, the response of species and functional traits were inconsistent across different rates of warming. A high tolerance to MHWs (represented by intercept values) did not necessarily correlate to a high tolerance, or a consistent trade-off, in longer term plasticity (indicated by slope values^43,44^). The equivocal evidence for a trade-off between tolerance and plasticity^44,45^ was thought to be due to experimental design, but our study shows that despite multiple traits affecting trade-offs within the Falklands assemblage there are consistent macrophysiological trade-offs across assemblages. This trade-off was clear despite the experiments being conducted over several months from spring through to the beginning of winter.

Species traits, such as mobility, feeding guild, or habitat, have been shown to influence the tolerance or plasticity of species. Sessile animals need to be able to withstand the conditions they experience in their environment, as once they settle, they must rely on their resilience to survive, and were therefore predicted to have a higher tolerance of MHWs. However, despite their ability to escape dangerous temperatures by moving, mobile animals can be more tolerant of warming^6^. Mechanistically this is thought to be a consequence of more active species having higher metabolic capacities and therefore greater aerobic scopes, which provide a physiological buffer against oxygen limitation, increasing thermal limits^46^. In our experiments, there was no difference between the three mobility groups, a similar finding to that of tropical marine ectotherms, whose acute tolerance was similarly uncorrelated with activity rates^4^. This may reflect a combination of two counteracting responses, with sessile organisms requiring resilience to survive MHWs while mobile animals tolerate higher acute temperatures because of their greater aerobic scopes. The slope of the sessile group was much steeper than the other two groups, indicating a reduced acclimation capacity. This is in line with previous empirical evidence^6^ and fits with the expectation of a physiological trade-off between thermal tolerance and plasticity^44^.

Different patterns between tolerance to MHWs and capacity to cope with long-term warming were observed between feeding guilds. Filter feeders, which had the highest tolerance to MHW, showed a trade-off with capacity to cope with longer-term warming (joint lowest with predators). While more research is required to understand the mechanisms underlying these differences between feeding guilds, energy supply is a key component of resilience, particularly to longer-term warming, as ectotherm energy requirements increase with temperature, as biochemical reactions speed up, resulting in a higher basal metabolic rate^1,46^. In the natural environment it is possible that different feeding guilds will have different capacities to increase energy intake relative to the increase in energy expenditure, altering their relative vulnerability. This is important to understand as the responses may be compounded by other impacts of ocean warming which will affect species survival. For example ocean warming is known to affect dissolved organic carbon, potentially including particle aggregation and therefore sinking^47,48^. This could clearly affect the amount of organic material reaching the seafloor and therefore the amount of energy available to detritivores, thereby indirectly affecting animal vulnerability to ocean warming, with potential cumulative or interactive effects. Vulnerability to ocean warming may be underestimated under laboratory conditions, when interactive stressors are unlikely to be included^49^ and the rates of warming employed in experiments are unlikely to test the full range of resilience mechanisms^16^. This is one of the strengths of the approach using different rates of warming, that it allows extrapolation from acute tolerance to over ecologically important longer-term scales of tolerance^6^. Temperature is just one of the environmental factors that is expected to change with changing climate, with additional effects such as ocean acidification, mixing, salinity and habitat loss amongst the factors that are expected to interact to affect species resilience^29^. Studies such as ours, therefore, form a component of the scientific evidence underpinning conservation and climate mitigation policy.

Our results indicated that, as predicted, kelp forest animals had lower resilience to longer-term heating than animals from outside kelp forests. It is likely that kelp forests have a stabilising effect on the water column environment within the forest. For example, kelp forests are a known natural protection for the coast against wave action and reducing mixing in the water column, which may potentially alter temperature gradients or variability^50^. The magnitude, variability and predictability of temperature plays a key role in the selection of species temperature tolerances^7^. If these factors differ for animals within and outside kelp forests, this may affect their responses to ocean warming. An opposite trend was observed in green sea urchins (*Stongylocentrotus droebachiensis*) from Canadian kelp forests, which had higher tolerances to ocean warming than urchins collected from urchin barrens^51^. Barrens, however, may be stressed for food or food quality, and disentangling the effects of thermal regime and energetics remains difficult. Further, the difference between the groups inside or outside the kelp forests may also be a result of the location in the water column. Most species collected from within the kelp forest were collected in shallower water, within the kelp canopy (except for *A. canaliculata*). The pelagic and kelp groups do not differ from each other in terms of resilience to MHW or capacity to cope with climate change, while benthic species had a greater capacity to cope with longer-term warming. Most benthic species were collected from outside the kelp forest, which likely conflates the effect of kelp on warming tolerance and capacity observed here. Obtaining these functional understandings are essential to provide the data requirements for ecosystem models to understand how marine food webs will change in response to climate change. Often, ecosystem models aggregate lower trophic-level organisms into a few functional groups because of the lack of information (on species, biomass, and functional responses to environmental change), but here we show that such aggregations can miss out on important biological differences, which in turn can have cascading consequences for higher trophic-level species that depend on these organisms for food.

We included small pelagic and large benthic *G. gregaria* to investigate ontogenetic effects. Their responses to the rates of warming were remarkedly different, with small *G. gregaria* having a higher temperature tolerance to MHW than large *G. gregaria*. This is in line with other empirical data that juveniles tolerate higher temperatures compared to larger, reproductive, individuals^6^. However, small *G. gregaria* showed a trade-off in CT_max_ between resilience to MHW and their capacity to cope with ocean warming in the long term, having a much reduced long-term physiological plasticity than that of the large *G. gregaria*. This is in contrast with previous findings, and an exception to the expectation of the oxygen and capacity limitation hypothesis^6^. Such ontogenetic mismatches in responses to ocean warming (regardless of size) has implications for the species and supports the importance of studying life history responses to climate change effects^52^. *G. gregaria* is especially important in the Falkland Islands marine food web, being consumed by a large range of taxa, from fishes to seabirds and marine mammals^28^. They are considered a wasp-waist species in the food web^53,54 ^meaning they have a disproportionate influence on the food-web dynamics. Thus, if *G. gregaria* population dynamics are affected by ocean warming via higher resilience of early pelagic life stages, or lower acclimations capacity in later stages, this will likely have consequences for the wider food web.

All species investigated in this study were collected from the East Falkland; similar species from the West Falklands may show different responses. The west Falkland Islands are warmer, as the Falkland Current strength is much lower here compared to the east, and the Argentine Drift brings warmer waters to the northwest Falklands^27,55–57^. More research is required to see if there are differences in assemblage or species responses between the east and west Falklands, and also how physiology varies across seasons and years. Any differences would have implications for the Falklands and wider marine Southwest Atlantic food web. Many large populations of higher trophic-level animals that prey on *G. gregaria* are predominately present on the west, including the largest breeding population of black-browed albatross, southern rockhopper penguins, and South American fur seals of the world^58–60^. Understanding regional variation in assemblage response towards short- and long-term ocean warming in coastal communities is required to improve predictions of ecosystem responses in the Falklands, and wider, marine ecosystem. This physiological trait-based understanding is essential if we are to build adaptive management and conservation strategies to cope with climate change impacts on the Falkland Islands.

### Global comparisons

This pattern of number of years until CTmax is reached is strongly correlated with the rate of predicted environmental warming, which is greater at low than high latitudes (Table S2). The relative tolerance of the Falkland Islands to ocean warming, is largely due to the slow predicted rate of warming. Based on this metric, the Falkland Islands is similar to other cold temperate areas such as New Zealand and Scotland (Fig. 4). The only two assemblages that had a higher number of years until CT_max_ would be reached are the two Antarctic assemblages, with the slowest rate of warming in the IPCC models.

However, the trade-off between short- and long-term resilience indicates that there are differences between the Falkland Islands and other cold temperate assemblages. It was expected that the response of the Falkland Islands marine assemblage to different rates of warming would be similar to that of southern New Zealand, a geographically similar southern high latitude fauna. Instead, however, the Falklands values fall well below the overall trend line, indicating they have a lower acclimation capacity (Fig. 3). Evidence from other assemblages indicates that the Falklands assemblage is likely to be subject to episodic, unpredictable, changes in temperature. It is only if changes in experienced temperature now are predictive of future temperatures (e.g. spring to summer seasonal changes), that acclimation capacity will be “beneficial” and it is only then that the adaptation of phenotypic flexibility is expected^61^. Acclimation is one of the elements of physiological capacity (tolerance, acclimation and adaptation^16^) that infers organism resilience against climate change^62^. A limited acclimation capacity is, therefore, likely to indicate vulnerability to future climate warming of the Falkland Islands assemblage.

The Falkland Current plays a significant role in driving upwelling around the Falklands, and the episodic nature of most upwelling systems may explain why the Falklands assemblage estimates were lower than predicted. The best known example is the upwelling off the coast of Peru^63^. The lower than expected estimates for the Peruvian assemblage (SHWT in Fig. 3) were attributed to the unpredictability in ocean temperatures as a result of El Niño-Southern Oscillation (ENSO) events which influences upwelling dynamics^7^. The nearshore Peruvian assemblage already suffers high mortality during El Niño events^63^, disruption that is only likely to get worse as climate continues to warm. The upwelling current and the variability in the position and strength of the Falklands Current likely results in a similar component of environmental unpredictability. While large-scale environmental change, including effects from ENSO, are understudied in the Falkland Islands, changes in SST related to ENSO have been correlated with negative impacts on Falklands populations of southern sea lions^64^, gentoo penguins^65,66^, southern rockhopper penguins^67^, black-browed albatross and Magellanic penguins^68^. The Falkland Current itself changes interannually in strength and direction, influencing both inshore and offshore environmental conditions, which are, for example, known to affect Patagonian squid feeding success^69^ and Patagonian toothfish recruitment success^70^. The Falkland Current is characterised by negative SST anomalies during El Niño events and positive SST anomalies during La Niña events, which is opposite to the fluctuations in SST anomalies in the Peruvian upwelling^71,72^. Thus, various factors may influence oceanographic dynamics around the Falkland Islands, resulting in a larger unpredictability in ocean temperatures which in turn influences the evolution of animal capacity to cope with ocean warming, similar to that observed in Peruvian assemblages. This influence of oceanographic dynamics around upwelling may also explain the differences between St. Helena and Ascension Island. The St. Helena assemblage also had a lower than expected short-term resilience, and this too could be the result of unpredictable changes in upwelling dynamics. The upwelling around St. Helena is influenced by the so-called ‘Benguela Niño’, which are dramatic interannual fluctuations in the Benguela upwelling system^73,74^. The assemblage from Ascension Island, which lies north of St. Helena, did not show such a trend. This may be the result of the limited number of species tested, but it may also be possible that the combination of the South Equatorial and Benguela Currents creates a local oceanographic regime that may provide more stability for Ascension Island compared to St Helena. If upwelling plays a role in the responses of these three assemblages, it may also explain why these three assemblages show the smallest difference in the number of years until reaching CT_max_ when MHW elevation was taken into account (14 years for Peru, 19 years for the Falklands, 26 years for St. Helena; all other assemblages have a difference of 59 years or more). A greater understanding of the impact of variability and predictability on species resilience, and the impacts of ecological traits on the trade-off between short- and long-term resilience to climate change are essential for the marine management of the response to MHW and long-term warming.

## Electronic supplementary material

Below is the link to the electronic supplementary material.

Supplementary Material 1

Supplementary Material 2

## Data Availability

The datasets generated during and/or analysed during the current study are available from the corresponding author on reasonable request.
